# Interfacial spin configurations and the microscopic origin of exchange bias at the IrMn/CoFeB interface

**DOI:** 10.1016/j.fmre.2026.01.003

**Published:** 2026-01-08

**Authors:** Jiahao Liu, Xingtai Chen, Jiaqi Lu, Weixiang Li, Zhaochun Liu, Yongzhuo Zhang, Peter Bencok, Paul Steadman, Wenqing Liu, Weisheng Zhao, Shouzhong Peng

**Affiliations:** a Fert Beijing Institute, School of Integrated Circuit Science and Engineering, Beihang University, Beijing 100191, China; b Hefei Innovation Research Institute, Beihang University, Hefei 230013, China; c National Key Laboratory of Spintronics, Hangzhou International Innovation Institute, Beihang University, Hangzhou 311115, China; d Department of Physics, Royal Holloway University of London, Egham, Surrey TW20 0EX, United Kingdom; e Beamtime I10, Diamond Light Source, Didcot OX11 0DE, United Kingdom; f Electrical Engineering Division, Department of Engineering, University of Cambridge, Cambridge CB3 0FA, United Kingdom

**Keywords:** Antiferromagnets, Exchange bias, X-ray magnetic circular dichroism, Spintronics, Magnetism

## Abstract

Exchange bias fields at antiferromagnet/ferromagnet (AFM/FM) interfaces play a crucial role in the performance of spintronic devices. Despite extensive research, the physical origin of exchange bias remains incompletely understood. In this study, we conduct a detailed investigation of a prototype AFM/FM interface widely used in spintronic applications, i.e., the IrMn/CoFeB interface. High-resolution synchrotron X-ray measurements reveal the existence of uncompensated Mn spins at the interface. While most of these spins are strongly coupled to the adjacent CoFeB layer, a small fraction remains pinned to the underlying IrMn underlayer. Element-specific X-ray magnetic circular dichroism hysteresis loops show that these pinned spins can be switched by increasing the annealing magnetic field. Furthermore, micromagnetic simulations indicate that an imbalance in the quantity of antiparallel pinned spins contributes to the observed variation in exchange bias. Overall, these findings offer important insights into the microscopic mechanisms governing exchange bias and its tunability.

## Graphical abstract

**Figure:**
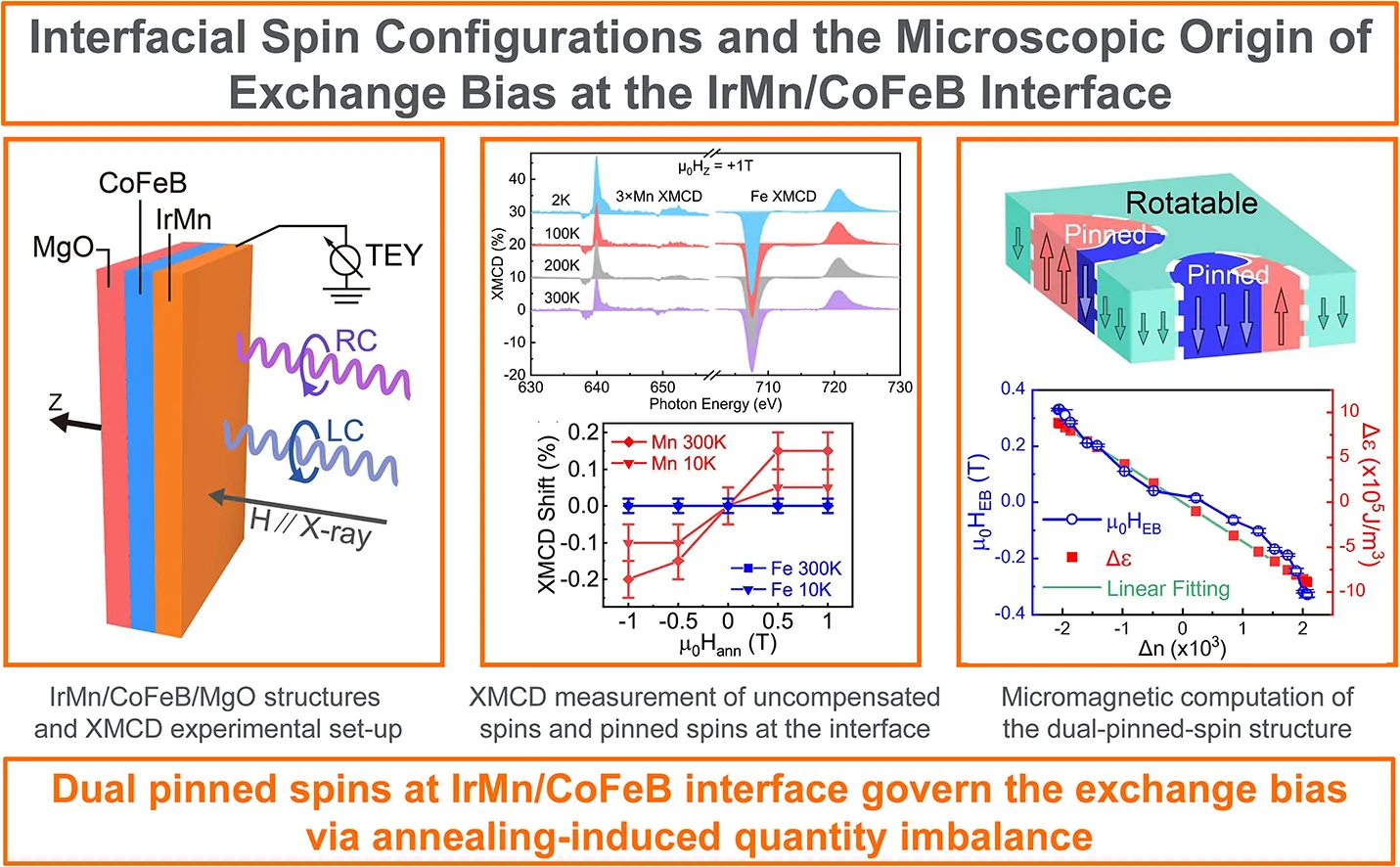

## 1 Introduction

The antiferromagnet/ferromagnet (AFM/FM) interface has attracted considerable attention due to its essential role in spintronics [[1], [2], [3], [4], [5], [6], [7], [8], [9], [10], [11], [12]]. Interfacial effects arising in AFM/FM structures, most notably exchange bias, have a significant impact on the performance of spintronic devices [[13], [14], [15], [16]]. Exchange bias refers to the shift of the magnetization hysteresis loop caused by interfacial coupling between the AFM and FM layers. In early work, this effect was employed to stabilize the magnetization of the pinned or reference layer in spin-valve structures and magnetic tunnel junctions [17]. Moreover, spin–orbit torque switching of perpendicular magnetization without an external magnetic field can be realized by introducing an in-plane exchange bias field [[18], [19], [20], [21], [22], [23], [24], [25], [26], [27], [28], [29], [30]]. A representative example is the IrMn/CoFeB/MgO heterostructure, which is widely used in magnetic tunnel junctions [[25], [26], [27]]. In this system, spin–orbit torque generated by IrMn enables switching of the CoFeB magnetization with the assistance of exchange bias from the IrMn/CoFeB interface, while the CoFeB/MgO structure provides strong perpendicular magnetic anisotropy and large tunneling magnetoresistance. In recent years, the electrical control of exchange bias via spin–orbit torque has been demonstrated [[31], [32], [33], [34], [35]], enabling the development of a novel storage prototype known as antiferromagnetic random-access memory. This technology exhibits strong resistance to magnetic field interference [11,[36], [37], [38]]. In such demanding applications, the exchange bias field has gained widespread adoption due to its durability, exceptional stability, and practical utility.

However, despite substantial research efforts, the physical mechanism underlying exchange bias at AFM/FM interfaces remains unresolved [31,32]. In 1956, Meiklejohn and Bean proposed that exchange bias originates from fully uncompensated AFM spins at the interface [39]. Later, in 1987, Mauri et al. introduced the planar domain-wall model to reconcile the quantitative discrepancies between theoretical predictions and experimentally measured exchange bias fields [40]. Malozemoff subsequently developed the random-field model to account for the influence of interface roughness and defects [41]. Experimentally, Ohldag et al. confirmed the presence of pinned spins in NiO/Co and other AFM/FM systems [42], and Roy et al. observed pinned AFM spins beneath the interface in FeF_2_/Co heterostructures [43]. Together, these established that the coupling between FM spins and pinned AFM spins is a key microscopic mechanism responsible for the exchange bias field. Nevertheless, the current physical understanding remains insufficient to fully describe how exchange bias fields can be manipulated. Recent studies on the electrical control of exchange bias via spin–orbit torque have shown that spin current can partially switch the exchange bias field [33,34,[44], [45], [46]]. This behavior is typically attributed to the formation of antiferromagnetic multidomains during the manipulation process, suggesting that the orientations of interfacial AFM spins, and their contributions to the exchange bias field, are more complex than previously assumed. Despite these developments, direct experimental evidence that clarifies these mechanisms remains limited.

In this study, we systematically investigate the physical mechanism of exchange bias and the interfacial spin configurations in the IrMn/CoFeB/MgO structure. X-ray magnetic circular dichroism (XMCD) is employed as a high-precision technique for probing and analyzing AFM/FM interfaces. First, XMCD measurements at different temperatures are conducted to examine the interfacial spin configuration in the IrMn/CoFeB/MgO system. Subsequently, element-specific XMCD hysteresis loops are measured to determine the orientations of AFM pinned spins in samples annealed under different magnetic fields. Based on the experimental findings, we propose a dual-pinned-spin interface model consisting of two types of AFM pinned spins with antiparallel orientations. Micromagnetic simulations show that variations in the exchange bias field are primarily governed by the quantitative imbalance between these pinned-spin populations. By integrating experimental analyses with micromagnetic simulations, we elucidate the composition of interfacial antiferromagnetic spins and their distinct contributions to the origin and modulation of the exchange bias field.

## 2 Materials and methods

### 2.1 Sample preparation

Thin films with the structure of Ta (2 nm)/Ir_20_Mn_80_ (5 nm)/Co_40_Fe_40_B_20_ (1 nm)/MgO (2.5 nm)/Al_2_O_3_ (5 nm) were deposited on thermally oxidized Si substrates by magnetron sputtering under vacuum. The bottom Ta layer serves as a buffer, while the top Al_2_O_3_ layer protects the multilayer stack from oxidation. Following deposition, the samples were annealed at 473 K for 30 min in a vacuum (<10^−7^ Torr) under out-of-plane magnetic fields ranging from +1 T to −1 T. X-ray diffraction measurements confirm a (111)-preferred orientation of the IrMn layer, as shown in Supplementary Fig. S1.

### 2.2 X-ray absorption spectrum (XAS) and XMCD measurements

XAS and XMCD measurements were conducted at the I10 beamline of the Diamond Light Source, UK. The experimental configuration is shown in Fig. 1a. The incident X-ray beam was oriented perpendicular to the sample plane and parallel to the applied external magnetic field, which was varied up to 1.1 T Measurements were performed over a temperature range of 2–300 K. Photon energies were scanned from 630 to 730 eV to cover the $L_{2}$ and $L_{3}$ edges of both Fe and Mn. The total XAS spectrum was obtained by summing the signals from left- and right-circularly polarized X-rays, $\left(I^{-}+I^{+}\right)$, where $I^{-}$ and $I^{+}$ represent the XAS spectra recorded with left- and right-handed helical polarization, (∼100% polarization), respectively. The XMCD signal was extracted as the difference between these spectra and normalized according to $\left(I^{-}-I^{+}\right)/\left(I^{-}+I^{+}\right)\;\times \;100\%$. The XMCD intensity was further normalized to the peak value of the corresponding XAS spectrum.

**Fig. 1:**
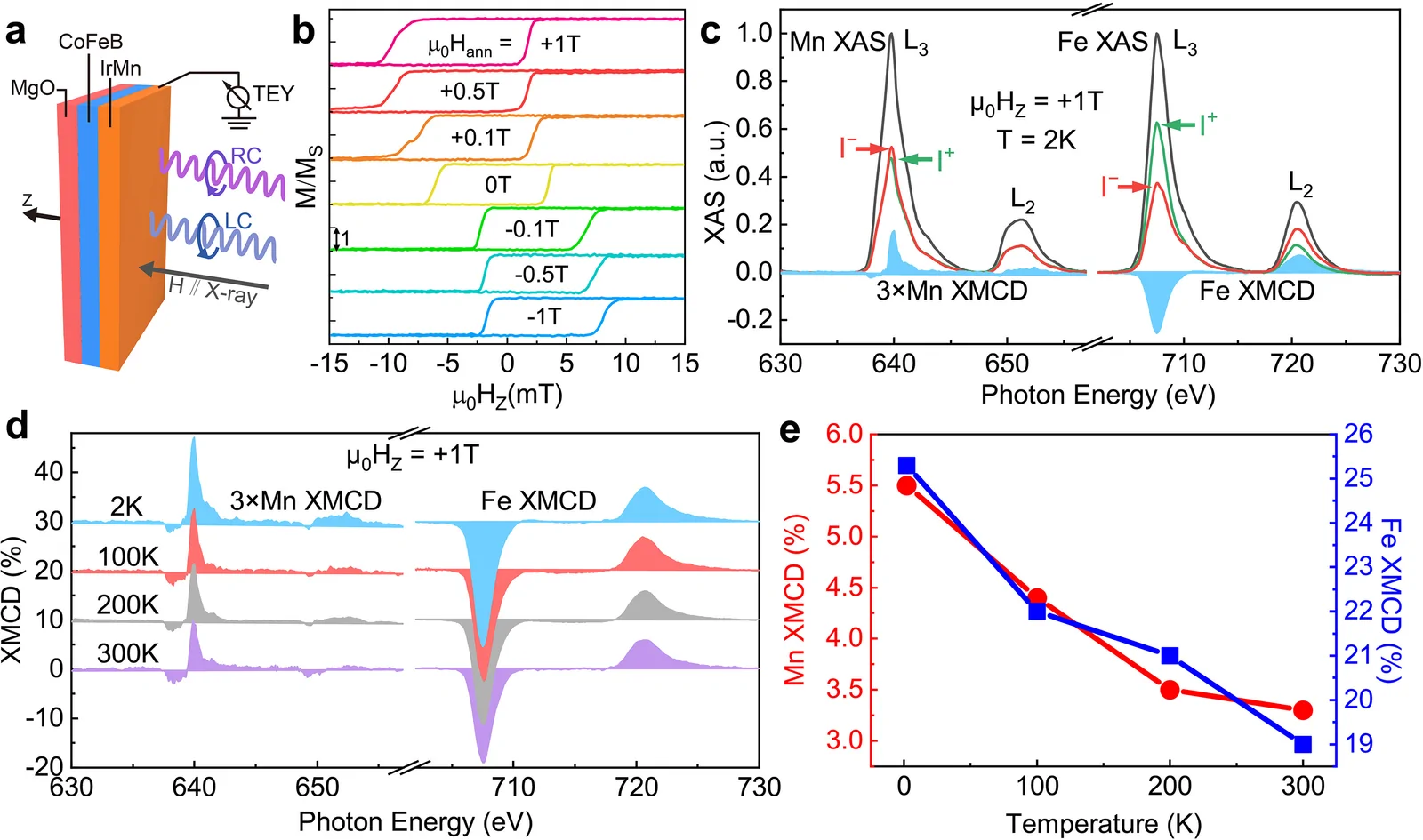
**XMCD characterization of IrMn/CoFeB/MgO multilayers annealed under *a*+1 T out-of-plane field.** (a) Schematic of the IrMn/CoFeB/MgO structure and the circularly polarized X-ray incidence direction. TEY denotes the total electron yield mode used to probe photoelectron signals from the sample interface. RC and LC represent right- and left-circularly polarized X-rays, respectively. (b) Perpendicular magnetization hysteresis loops of IrMn/CoFeB/MgO films after annealing under different fields at 473 K. (c) XAS and XMCD spectra of Mn and Fe measured at 2 K under *a* + 1 T external field. (d) Fe and Mn XMCD spectra under *a* + 1 T field at different temperatures. (e) Temperature dependence of the normalized L_3_ dichroism.

### 2.3 Micromagnetic simulations

Micromagnetic simulations were performed using Muamx^3^, a GPU-accelerated simulation platform [[47], [48]]. The simulated bilayer structure has lateral dimensions of 500 nm and consists of a 1.5-nm thick FM film and a 0.5-nm thick AFM film. Only the interfacial uncompensated AFM spins are included in the model, while the antiparallel compensated spin arrangement is excluded, as the microscopic origin of exchange bias is attributed to uncompensated interfacial AFM moments [32]. The simulation region is discretized into 128 × 128 × 4 cells, ensuring a cell size below 4 nm. Detailed micromagnetic simulation parameters are provided in the Supplementary Materials.

## 3 Results and discussion

### 3.1 XMCD characterization of uncompensated spins

After annealing under different magnetic fields, the out-of-plane magnetization curves (M–H loops) were measured using a vibrating sample magnetometer. As shown in Fig. 1b, the multilayer exhibits perpendicular magnetic anisotropy along with an out-of-plane exchange bias field. The magnitude of the exchange bias increases monotonically with the annealing field and saturates at ±1 T.

To elucidate the mechanism behind the variation of the exchange bias and the interfacial spin configuration, we performed XAS measurements on samples annealed under *a* + 1 T out-of-plane magnetic field. The samples were first cooled from room temperature to 2 K in zero magnetic field. As illustrated in Fig. 1a, XAS measurements were then carried out with the incident X-rays and an external +1 T magnetic field applied perpendicular to the film plane. Fig. 1b shows the corresponding XAS and XMCD spectra for Mn and Fe at 2 K. The Mn XMCD signal indicates the presence of net Mn moments at the IrMn/CoFeB interface, likely arising from locally uncompensated antiferromagnetic order. The XMCD amplitude of Mn (5%) is much weaker than that of Fe (25%), suggesting that the number of uncompensated Mn spins is relatively small. The net interfacial Mn spins may originate from two mechanisms: (1) spin canting induced by the adjacent ferromagnetic layer, as described in the spin-flop model [12], and (2) local ferromagnetic ordering resulting from interfacial defects or lattice imperfections [49,50]. Notably, the Mn XMCD signal at the L_3_ edge has the opposite sign to that of Fe, confirming strong antiferromagnetic coupling between Fe and Mn spins, which is strong enough that an external +1 T field cannot overcome the antiparallel alignment. Subsequently, XMCD measurements were performed as the sample was heated to 100, 200, and 300 K in zero magnetic field. As shown in Fig. 1c, the XMCD signals of Mn and Fe increase as the temperature decreases, and the Mn signal remains opposite in sign to the Fe signal at all temperatures. The corresponding XAS spectra for Mn and Fe at different temperatures are provided in Fig. S2. Fig. 1d summarizes the temperature dependence of the Fe and Mn XMCD intensities at the L_3_ edge, confirming the presence of uncompensated Mn spins at the interface and their persistent antiferromagnetic coupling with Fe spins.

### 3.2 XMCD characterization of pinned spins

To further investigate the composition of the uncompensated Mn spins, XMCD measurements were performed with external magnetic fields swept between +20 and −20 mT. Fig. 2 presents the element-specific XMCD hysteresis loops for samples annealed under +1 T, −1 T, and zero fields. For Fe (Fig. 2a), the XMCD saturation values under large magnetic fields correspond to the positive and negative saturation magnetizations, $M_{Fe}^{+}$ and $M_{Fe}^{-}$, respectively. Notably, the Fe XMCD hysteresis loops show no vertical offset, indicating that all Fe spins are fully switchable under the applied ±20-mT magnetic field. Co XMCD hysteresis loops exhibit magnetization switching behavior consistent with that of Fe, as shown in Supplementary Fig. S3. The corresponding Mn XMCD hysteresis loops are displayed in Fig. 2b. Because the Mn XMCD signal originates from interfacial uncompensated spins, the positive and negative saturation values are denoted as $M_{uncomp}^{+}$ and $M_{uncomp}^{-}$, respectively. The Mn XMCD signal exhibits an opposite sign relative to Fe, confirming that the majority of the uncompensated Mn spins remain antiferromagnetically coupled to the Fe layer. Furthermore, this fraction of uncompensated Mn spins reverses together with the ferromagnetic layer, displaying ferromagnetic-like switching behavior; thus, these spins are categorized as rotatable spins.

**Fig. 2:**
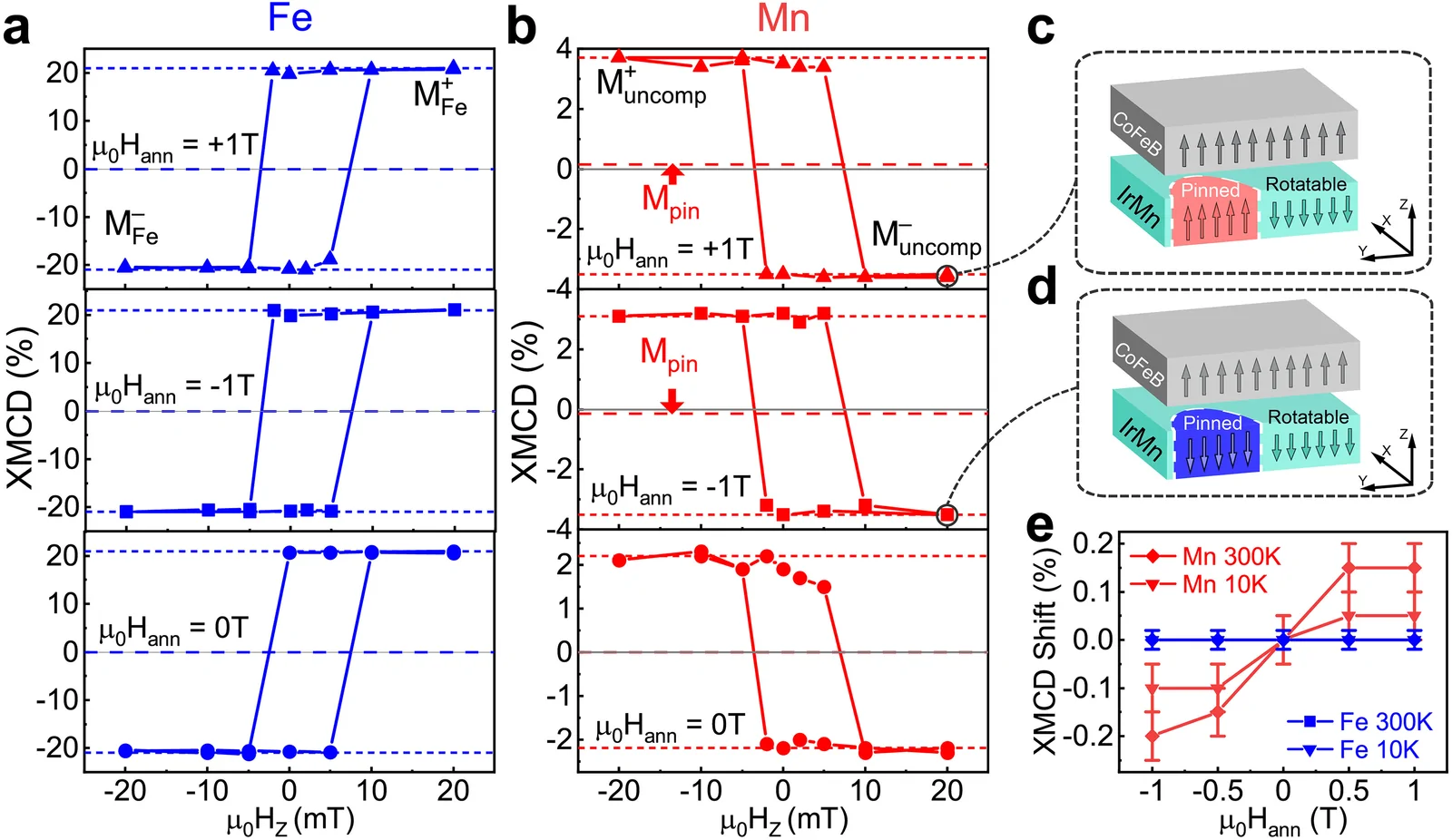
**Element-specific XMCD hysteresis loops measured on samples annealed under different magnetic fields.** (a) Fe and (b) Mn XMCD hysteresis loops measured at 300 K for samples annealed under +1, −1T, and zero fields. Schematic illustrations of the interfacial CoFeB and IrMn spin orientations after annealing under (c) *a* + 1 T field and (d) *a* − 1 T field. Green, red/blue, and gray arrows indicate Mn rotatable spins, Mn pinned spins, and CoFeB spins, respectively. (e) XMCD vertical shift of Mn and Fe as a function of annealing field measured at 10 and 300 K, with an estimated uncertainty of <0.05%. Mn exhibits a clear annealing field–dependent shift, whereas no measurable shift is observed for Fe.

Importantly, for the sample annealed under *a* + 1 T field, the Mn XMCD signal at −20 mT is larger than that at +20 mT, indicating that $M_{uncomp}^{+}$ > $M_{uncomp}^{-}$. This imbalance results in a positive vertical shift of the Mn XMCD hysteresis loop. This observation demonstrates that not all uncompensated Mn spins can be switched by the applied field; if they could, the condition $M_{uncomp}^{+}+M_{uncomp}^{-}\;=\;0$ would hold, and no vertical shift would appear in the hysteresis loop, as is observed for Fe. Accordingly, a fraction of the uncompensated Mn spins is pinned at the IrMn/CoFeB interface, which can be quantified as $M_{pin}\;=\;\left(M_{uncomp}^{+}+M_{uncomp}^{-}\right)/2$. Thus, the total uncompensated Mn spins consist of both rotatable and pinned components (Fig. 2c), expressed as $M_{uncomp}=M_{rot}+M_{pin}$. Furthermore, the sample annealed under a –1 T field exhibits a negative vertical shift in the Mn XMCD hysteresis loop, in contrast to the positive shift observed for the +1 T field–annealed sample. This reversal arises from the opposite orientation of the pinned spins (Fig. 2c,d), which is determined by the direction of the annealing field.

For the sample annealed under zero field, the Mn XMCD hysteresis loops show no vertical shift. In this case, the IrMn/CoFeB interface is likely to host pinned spins in opposite directions, a configuration we refer to as the dual-pinned-spin model. Without an applied annealing field to induce an imbalance, the numbers of positive and negative pinned domains are expected to be equal. Consequently, the antiparallel pinned spins generate XMCD signals of equal magnitude but opposite polarity, resulting in no net vertical shift in the Mn XMCD hysteresis loop. Fig. 2e shows the dependence of the XMCD shift on the annealing field for both Mn and Fe. While the Fe XMCD hysteresis loops exhibit no shift, the Mn XMCD hysteresis loops display clear annealing field–dependent shifts at 10 and 300 K: upward shifts under positive annealing fields and downward shifts under negative fields. This behavior provides direct evidence for the selective switching of Mn pinned spins. Additional Mn XMCD spectra and hysteresis loops corresponding to Fig. 2e are provided in Fig. S4.

### 3.3 Micromagnetic simulations of dual-pinned-spin configurations

Building on the dual-pinned-spin model, the relationship between pinned spins and the exchange bias field can be further examined. Exchange bias is understood as a unidirectional anisotropy, suggesting that the pinned spins, which do not switch under an external magnetic field, serve as the primary source of the exchange bias field, whereas the rotatable spins do not contribute. As described above, however, the interface hosts dual oppositely oriented pinned spins. To clarify their distinct contributions to the magnitude and direction of the exchange bias, we conducted micromagnetic simulations of an AFM/FM interface, explicitly resolving the interactions between interfacial spin populations and the resulting exchange bias.

The micromagnetic model comprises three components: FM spins, AFM pinned spins, and AFM rotatable spins. Spin orientations are initially randomized. The structure is then subjected to annealing simulations at 473 K under external out-of-plane fields ranging from −6 to +6 T, during which the FM and interfacial AFM spins align with the applied field. The system is subsequently cooled to 300 K and allowed to relax to equilibrium. Throughout this process, the FM and pinned spins retain their orientations, while the rotatable spins spontaneously reverse alignment, driven by strong antiferromagnetic exchange coupling with the FM layer, consistent with the XMCD observations in Fig. 1b.

Fig. 3 illustrates the interfacial AFM spin configurations after annealing, along with corresponding cross-sectional schematics. The regions highlighted by white dashed circles represent AFM pinned spins, with red spins aligned along +z and blue spins along −z, while the green areas correspond to AFM rotatable spins. Following ±6 T field annealing, nearly all pinned spins reorient to align with the direction of the applied field (Fig. 3a,e). Under intermediate fields (e.g., ±3 T), only a portion of the pinned spins reconfigures to adopt field-parallel orientations (Fig. 3b,d). Notably, zero-field annealing produces a symmetric distribution of pinned spins oriented in opposite directions (Fig. 3c). Extended annealing simulations over the ±6 T field range (Fig. S5) further demonstrate the tunable reconfiguration of dual pinned spins at the AFM/FM interface, consistent with the XMCD experimental observations.

**Fig. 3:**
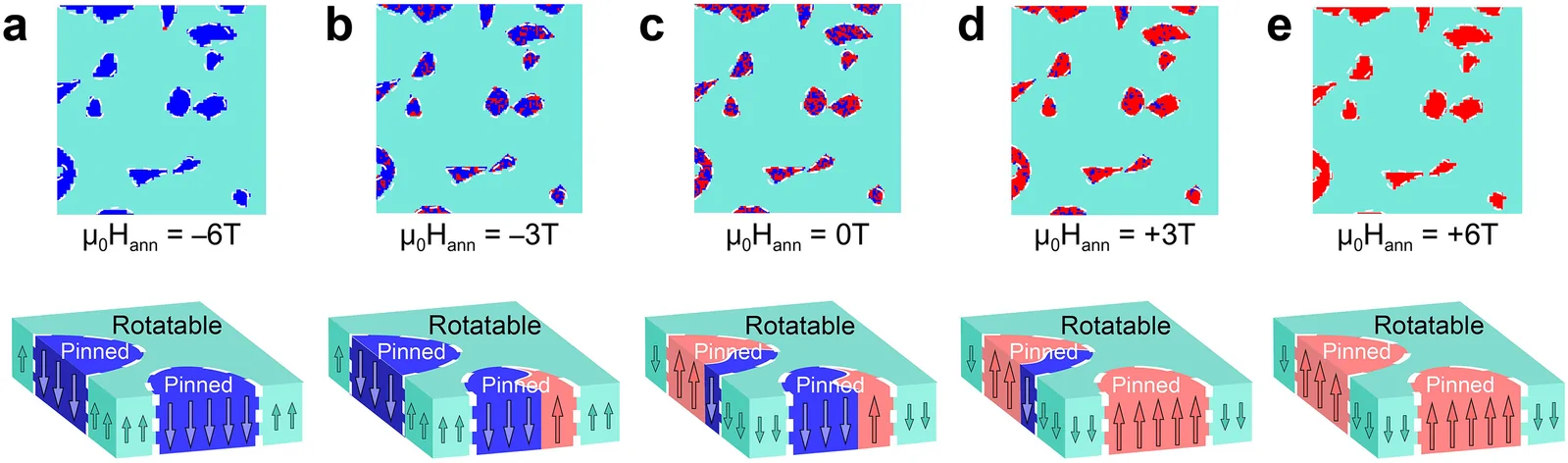
**Snapshots of interfacial AFM spin configurations from micromagnetic simulations of exchange bias.** Spin arrangements and corresponding cross-sectional schematics are shown after annealing under (a) −6, (b) −3, (c) 0, (d) +3, and (e) +6 T magnetic fields. Pinned spins are highlighted with white dashed circles. Red and blue spins represent pinned AFM spins aligned along +z and −z, respectively, while green regions correspond to AFM rotatable spins.

Following the field annealing simulations, magnetic hysteresis loops were simulated under out-of-plane fields swept between −0.04 and +0.04 T at 300 K. The exchange bias field, determined from the horizontal shift of hysteresis loops, increases monotonically with annealing field strength (Fig. 4a), while samples annealed under zero field exhibit a negligible exchange bias. To elucidate the mechanisms underlying the variation of the exchange bias, we quantified both the population imbalance of antiparallel pinned spins and the corresponding exchange bias field after annealing under different magnetic fields (Fig. 4b). The difference in the number of antiparallel pinned spins is defined as $\Delta n\;=\;(n_{+}-n_{-})$, where $n_{+}$ and $n_{-}$ represent the numbers of +z and −z pinned spins, respectively. Remarkably, the exchange bias field and Δn show identical trends as a function of annealing field: both approach zero after zero-field annealing, increase monotonically with the field strength, and eventually saturate at high fields. These results indicate that the exchange bias field is intrinsically determined by the configuration of dual pinned spins. In the following, we discuss the specific contributions of the dual-pinned-spin populations to the origin and modulation of the exchange bias.

**Fig. 4:**
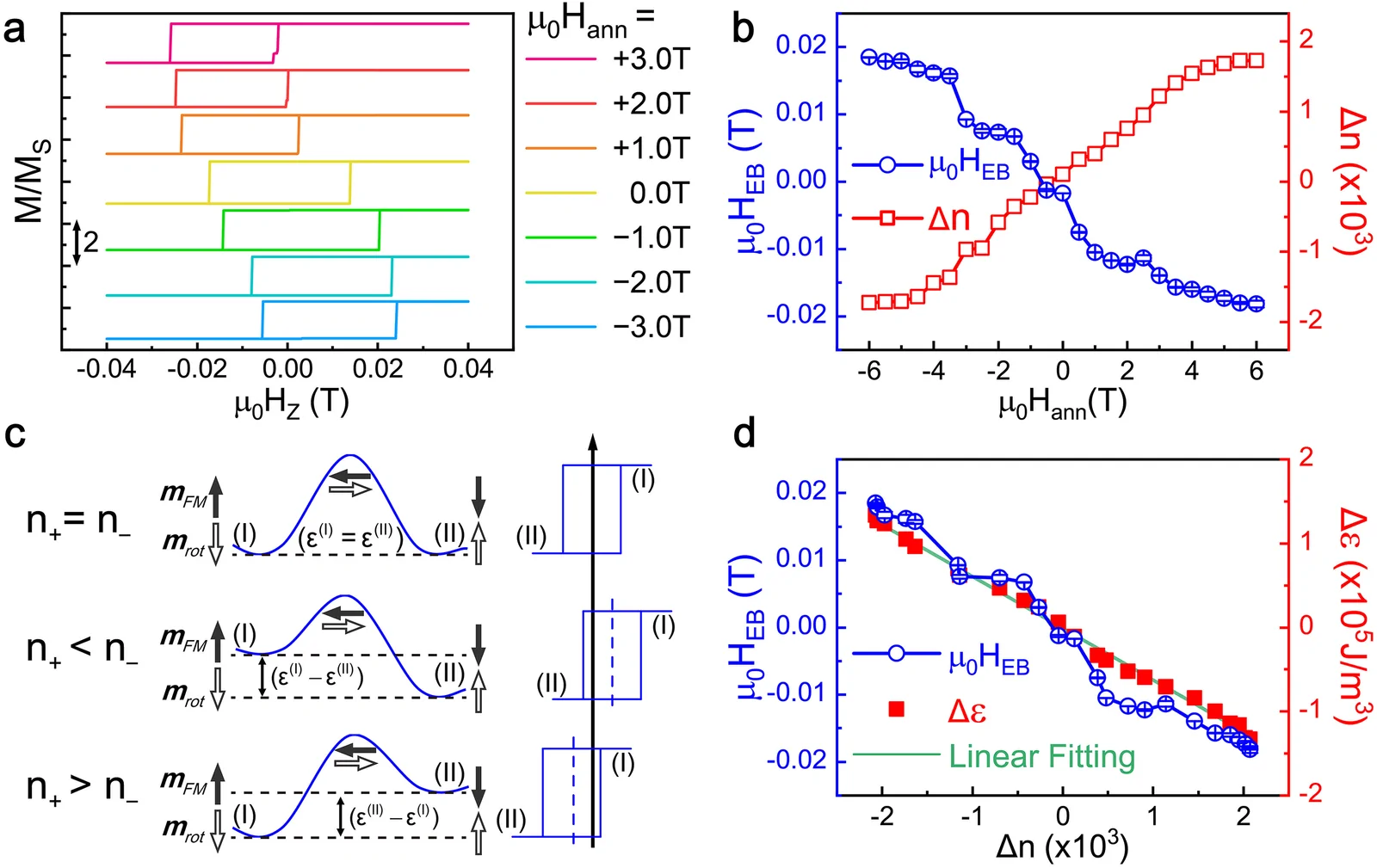
**Exchange bias fields and dual pinned-spin differences extracted from micromagnetic simulations.** (a) Simulated magnetic hysteresis loops after annealing under different out-of-plane fields. (b) Dependence of the exchange bias field and the population difference of antiparallel AFM pinned spins (Δn) on the annealing field. (c) Schematic illustrating the origin and variation of exchange bias based on the dual-pinned-spin model. Black and white arrows indicate the orientations of FM spins and AFM rotatable spins, respectively, while $n_{+}$ and $n_{-}$ denote the numbers of +z and −z pinned spins, respectively. $\varepsilon^{\left(/\right)}$ and $\varepsilon^{\left(//\right)}$ represent the energy densities of states (Ⅰ) and (Ⅱ), respectively. (d) Exchange bias field and interfacial energy density difference (Δε) between states (Ⅰ) and (Ⅱ) as a function of Δn. Exchange bias values are averaged over five simulations, with error bars representing the standard deviation.

### 3.4 Theoretical analysis

We examine the evolution of the system energy density under field-driven reversal. As illustrated in Fig. 4c, in state (Ⅰ) under an external field of +0.04 T, the FM spins align along +***z*** while the rotatable spins orient along −***z*** ($m_{FM}\;=\;+\hat{z}$, $m_{rot}\;=\;-\hat{z}$**)**, whereas in state (Ⅱ) under −0.04 T, these orientations are reversed ($m_{FM}\;=\;-\hat{z}$, $m_{rot}\;=\;+\hat{z}$**)**. The energy density difference between these two states can be expressed as $\Delta \varepsilon =\varepsilon^{\left(I\right)}-\varepsilon^{\left(II\right)}\;=\;-\frac{4\Delta V}{V}\left(n_{+}-n_{-}\right)\left(A_{ex}^{pin-FM}-A_{ex}^{pin-rot}\right)$, where ΔV is the simulated cell volume, V is the total system volume, and $A_{ex}$ denotes the exchange stiffness constant (see Supplementary Materials for details). This relation indicates that Δε is linearly correlated with $\Delta n\;=\;(n_{+}-n_{-})$. As shown in Fig. 4c, a balanced pinned-spin population (Δn=0) yields identical energy densities for pre- and post-reversal states (Δε=0), resulting in no exchange bias. In contrast, when −z oriented pinned spins outnumber +z spins (Δn<0), the energy density of state (Ⅰ) exceeds that of state (Ⅱ) (Δε>0). This energy asymmetry increases the Zeeman energy barrier for FM spin reversal from −z to +z, producing a positive unidirectional shift in the hysteresis loop, i.e., a positive exchange bias. Conversely, *a* + ***z***-dominant pinned-spin configuration (Δn>0) reverses the energy asymmetry (Δε<0) and gives rise to a negative exchange bias. Importantly, as shown in Fig. 4b, the quantity difference Δn can be controlled via annealing fields, which in turn tunes Δε and modulates the exchange bias. Extracted values of Δε from annealing simulations (Fig. 4d) exhibit a monotonic increase with increasing Δn, driving the enhancement of the exchange bias field. Therefore, the micromagnetic simulations demonstrate that both the direction and magnitude of the exchange bias are governed by the population imbalance of dual pinned spins with antiparallel orientations, providing a quantitative explanation for the experimentally dependence of exchange bias on annealing field.

## 4 Conclusion

We have performed a comprehensive study of the physical mechanisms underlying exchange bias and the interfacial spin configurations in the IrMn/CoFeB/MgO system. Element-specific XMCD measurements reveal that the interfacial uncompensated Mn spins, whose magnitude increases with decreasing temperature, consist of both rotatable and pinned spins. Analysis of XMCD hysteresis loops demonstrates that the pinned spins at the interface can be reoriented by varying the annealing field, leading us to propose a dual-pinned-spin interface model, in which two components of AFM pinned spins with antiparallel orientations coexist. Micromagnetic simulations further show that the population imbalance between these two components, which can be tuned by annealing fields, drives variations in the interfacial energy density difference. This energy asymmetry, in turn, dictates the magnitude and direction of the exchange bias field. Collectively, these findings provide a microscopic understanding of exchange bias and offer valuable insights for its controlled manipulation in AFM-based spintronic devices.

## CRediT authorship contribution statement

**Jiahao Liu:** Writing – review & editing, Writing – original draft, Validation, Formal analysis, Data curation. **Xingtai Chen:** Writing – review & editing, Investigation, Formal analysis, Data curation. **Jiaqi Lu:** Writing – review & editing, Investigation, Formal analysis. **Weixiang Li:** Investigation, Formal analysis. **Zhaochun Liu:** Investigation, Formal analysis. **Yongzhuo Zhang:** Validation. **Peter Bencok:** Formal analysis, Data curation. **Paul Steadman:** Formal analysis, Data curation. **Wenqing Liu:** Writing – review & editing, Supervision, Resources, Formal analysis. **Weisheng Zhao:** Supervision, Resources, Project administration, Funding acquisition. **Shouzhong Peng:** Writing – review & editing, Supervision, Funding acquisition, Formal analysis, Conceptualization.

## Declaration of competing interest

The authors declare that they have no conflicts of interest in this work.

## Appendix Supplementary materials

Image, application 1
