# Supplementary material for: Interfacial spin configurations and the microscopic origin of exchange bias at the IrMn/CoFeB interface
Source: Fundam Res. 2026 Jan 8;6(5):2891–7. doi: 10.1016/j.fmre.2026.01.003 (PMC13644517; doi:10.1016/j.fmre.2026.01.003)
Supplement: Supplementary file 1 [file mmc1.docx]

**Supplementary** **Material**

**Interfacial Spin Configurations and the Microscopic Origin of Exchange Bias at the IrMn/CoFeB Interface**

**This PDF file includes:**

Supplementary Text

Fig. S1. X-ray diffraction scan of the Ta (2 nm)/Ir_20_Mn_80_ (5 nm)/Co_40_Fe_40_B_20_ (1 nm)/MgO (2.5 nm)/Al_2_O_3_ (5 nm) sample

Fig. S2. The XAS spectra of Mn and Fe at different temperatures.

Fig. S3. Element-specific Co XMCD hysteresis loops under varying field-annealing conditions.

Fig. S4. Mn XMCD hysteresis loops and corresponding Mn XMCD spectra.

Fig. S5. Snapshots of the interfacial AFM in micromagnetic simulations of exchange bias.

Supplementary Text

**X-ray diffraction scan of the Ta (2 nm)/Ir_20_Mn_80_ (5 nm)/Co_40_Fe_40_B_20_ (1 nm)/MgO (2.5 nm)/Al_2_O_3_ (5 nm) sample**

X-ray diffraction θ - 2θ scan of the IrMn/CoFeB/MgO stack was conducted to determine the crystal orientation of IrMn layer, which is (111)-preferred, as shown in Fig. S1.

**X-ray magnetic circular dichroism hysteresis loops**

At different temperatures from 2 K to 300 K, XAS measurements are performed with incident X-rays and external +1 T magnetic fields perpendicular to the film plane. Fig. S2 shows the XAS and XMCD results of Mn and Fe. Mn XMCD results demonstrate that there are net Mn moments at the IrMn/CoFeB interface, which exhibit clear temperature dependence and maintain antiferromagnetic coupling with Fe spins.

Additionally, we performed Co-L_3_ edge XMCD measurements under identical experimental conditions to the Fe-XMCD data, as shown in Fig. S3. The Co-XMCD loops exhibit nearly identical switching fields to the Fe-XMCD loop (Fig. 2), indicating that Co and Fe moments are rigidly coupled within the CoFeB layer, undergoing synchronized magnetization reversal. Crucially, the Co-XMCD loop shows no vertical shift in contrast to Mn-loops, mirroring the behavior observed in the Fe-XMCD loop. This confirms that all the Co spins can be switched by the external field of ±20 mT at the IrMn/CoFeB interface, consistent with Fe.

Fig. S4 shows Mn X-ray magnetic circular dichroism (XMCD) hysteresis loops and Mn XMCD spectra measured in samples annealed under different out-of-plane magnetic fields between +1 T and $-$1 T. The saturation values in XMCD hysteresis loops are obtained from the L_3_ edge in XMCD spectra measured when the structure reaches the saturated magnetization state under a large magnetic field. The coercive field measured at 10 K is larger than that measured at 300 K since the rotatable Mn spins rotate with the CoFeB layer simultaneously, whose perpendicular magnetic anisotropy is enhanced at low temperature. The vertical shift in Mn XMCD loops, as an indicator of Mn pinned spin XMCD signals, is determined by the annealing field, corresponding to the Mn pinned spins as a function of annealing fields shown in Fig. 2.

**Antiferromagnet interface in annealing simulations**

In micromagnetic simulations, the granular structure of the AFM/FM interface is generated using a 3D Voronoi tessellation [1]. Both the FM and AFM layers are divided into 10 nm grains, which are randomly generated and remain unchanged during the simulation. Magnetic anisotropy constants according to previous works [2] are adopted for FM spins ($4.05\times10^{5}J/m^{3}$) and AFM rotatable spins ($6\times10^{5}J/m^{3}$). A larger magnetic anisotropy constant is set for AFM pinned spins ($7.5\times10^{6}J/m^{3}$) [3], with the easy axis along the z-axis. Exchange constants are set to $1\times10^{-11}J/m$ for FM spins and AFM rotatable spins, $6\times10^{-12}J/m$ for AFM pinned spins. The Landau-Lifshitz damping constant $\alpha$ is 0.05 and saturation magnetization $M_{S}$ is $800 KA/m$. Coupling between different spins is realized by setting different exchange stiffness $A_{ex}$. Since XMCD experiments reveal the antiferromagnetic coupling between rotatable AFM spins and FM spins, we set $A_{ex}^{rot-FM}=-0.15\times10^{-12}J/m$ and $A_{ex}^{rot-pin}=-3\times10^{-12}J/m$. The interfacial AFM pinned spins couple ferromagnetically to FM spins, set as $A_{ex}^{FM-pin}=1.2\times10^{-15}J\cdot m^{-1}$. During the hysteresis loops simulation, the energy of the whole system is minimized by the steepest conjugate gradient method. Between steps in 0.0002 T increments, the maximum change of magnetization required to stop energy minimizing is set to $1\times10^{-6}$.

Annealing is simulated under different fields between +6 T and $-$6 T, with the temperature set to 473K. Afterwards, the temperature is set to 300 K, and the system relaxes to reach an equilibrium state by solving the Landau-Lifshitz-Gilbert equation. The influence of temperature on magnetic moments is performed by fluctuating fields [1]. The antiferromagnet (AFM) interfaces after annealing under different fields are shown in Fig. S5. The areas marked by the white dashed circles are composed of AFM pinned spins (red spins align in $+\boldsymbol{z}$ while blue spins align in $-\boldsymbol{z}$.), with the other green areas representing AFM rotatable spins. Field-induced spin reconfiguration reveals a distinct correlation: higher annealing fields progressively align pinned spins with the field direction, leading to the orientation uniformity at saturation.

**Theoretical analysis of the dependence of exchange bias on pinned spins**

In Fig. 4, we focus on the energy density evolution of interface before and after field-driven reversal of FM and AFM rotatable spins, namely state (Ⅰ) $\boldsymbol{m}_{FM}=+\hat{\boldsymbol{z}}$, $\boldsymbol{m}_{rot}=-\hat{\boldsymbol{z}}$ and state (Ⅱ) $\boldsymbol{m}_{FM}=-\hat{\boldsymbol{z}}$, $\boldsymbol{m}_{rot}=+\hat{\boldsymbol{z}}$, while pinned AFM spins remain fixed. The energy density $\varepsilon$ of this magnetic system can be expressed as $\varepsilon=\varepsilon_{demag}+\varepsilon_{anis}+\varepsilon_{exch}$, where the three items (from left to right) represent the contribution from magnetostatic fields, magnetic anisotropy, and exchange fields, respectively. The demagnetization energy density is calculated by $\varepsilon_{demag}=-\frac{\mu_{0}}{2}\boldsymbol{m}\cdot\boldsymbol{B}_{demag}$ [1], where $\boldsymbol{B}_{demag}$ are the magnetostatic field. As the system is regarded as a thin film multilayer, the direction of magnetostatic fields is in-plane. Since all spins in the equilibrium state align in either $+\hat{\boldsymbol{z}}$ or $-\hat{\boldsymbol{z}}$, demagnetization energy density is identical in states (Ⅰ) and (Ⅱ). Magnetic anisotropy energy density can be expressed by $\varepsilon_{anis}=-K_{u1}(\hat{\boldsymbol{z}}\cdot\boldsymbol{m})^{2}$ [1], where $K_{u1}$ is the first-order uniaxial anisotropy constant. Thus $\varepsilon_{anis}=-K_{u1}$ in both (Ⅰ) and (Ⅱ). The direction and strength of thermal fluctuating fields are random and independent of spin configuration. In addition, the influence of temperature on magnetic moments is performed by fluctuating fields [1]. Therefore, the energy density difference $\varepsilon$ between the two states should be attributed to $\varepsilon_{exch}$. The exchange energy density consists of six components referring to the exchange coupling between different interfacial spins respectively:

$\varepsilon_{exch}=\frac{2\Delta V}{V}(\sum\varepsilon_{exch}^{FM-FM}+\sum\varepsilon_{exch}^{rot-rot}+\sum\varepsilon_{exch}^{pin-pin}+\sum\varepsilon_{exch}^{FM-rot}+\sum\varepsilon_{exch}^{pin-FM}+\sum\varepsilon_{exch}^{pin-rot})$.

$\Delta V$ denotes the cell volume and $V$ is the system volume. Because the ferromagnet (FM) spins are oriented in the same direction in state (Ⅰ) or (Ⅱ), exchange energy density between adjacent FM spins is calculated as $\varepsilon_{exch}^{FM-FM}=-A_{ex}^{FM-FM}\boldsymbol{m}_{FM1}(\boldsymbol{m}_{FM2}-\boldsymbol{m}_{FM1})=0$, where $A_{ex}$ refers to the exchange stiffness constant between spins. For the same reason, $\varepsilon_{exch}^{rot-rot}=0$. The distribution and direction of pinned spins remain the same, and thus $\sum\varepsilon_{exch}^{pin-pin}$is identical in states (Ⅰ) and (Ⅱ). Moreover, $\varepsilon_{exch}^{FM-rot}=-A_{ex}^{FM-rot}\boldsymbol{m}_{FM}(\boldsymbol{m}_{FM}-\boldsymbol{m}_{rot})=-2A_{ex}^{FM-rot}$ would hold in both (Ⅰ) and (Ⅱ), thus $\varepsilon_{exch}^{FM-rot}$also remains identical. Consequently, the energy density difference $\varepsilon$ between (Ⅰ) and (Ⅱ) is attributed to $\varepsilon_{exch}^{pin-FM}$ and $\varepsilon_{exch}^{pin-rot}$. Regarding $n_{+}$ and $n_{-}$ as the number of $+\boldsymbol{z}$ and $-\boldsymbol{z}$ pinned spins respectively, $\sum\varepsilon_{exch}^{pin-FM}$ is calculated as:$\sum\varepsilon_{exch}^{pin-FM}=\sum_{1}^{n_{+}} 2\varepsilon_{exch}^{(+pin)-FM}+\sum_{1}^{n_{-}} 2\varepsilon_{exch}^{(-pin)-FM}=-n_{+}A_{ex}^{pin-FM}\boldsymbol{m}_{FM}\cdot(\boldsymbol{m}_{+pin}-\boldsymbol{m}_{FM})-n_{-}A_{ex}^{pin-FM}\boldsymbol{m}_{FM}\cdot(\boldsymbol{m}_{-pin}-\boldsymbol{m}_{FM})$, where $\varepsilon_{exch}^{(+pin)-FM}$ denotes the exchange energy density between FM spins and $+\boldsymbol{z}$ pinned spins, while $\varepsilon_{exch}^{(-pin)-FM}$ refers to that between FM spins and $-\boldsymbol{z}$ pinned spins. On the other hand, $\sum\varepsilon_{exch}^{pin-rot}$ can also be expressed as: $\sum\varepsilon_{exch}^{pin-rot}=-n_{+}A_{ex}^{pin-rot}\boldsymbol{m}_{rot}\cdot(\boldsymbol{m}_{+pin}-\boldsymbol{m}_{rot})-n_{-}A_{ex}^{pin-rot}\boldsymbol{m}_{rot}\cdot(\boldsymbol{m}_{-pin}-\boldsymbol{m}_{rot})$. Therefore, the system energy density difference can be quantified as $\varepsilon^{(Ⅰ)}-\varepsilon^{\left( Ⅱ \right)}=\frac{2\Delta V}{V}\left[ \left( 2n_{-}A_{ex}^{pin-FM}+2n_{+}A_{ex}^{pin-rot} \right)-\left( 2n_{+}A_{ex}^{pin-FM}+2n_{-}A_{ex}^{pin-rot} \right) \right]=-\frac{4\Delta V}{V}(n_{+}-n_{-})(A_{ex}^{pin-FM}-A_{ex}^{pin-rot})$. Since the exchange stiffness constant $A_{ex}^{pin-FM}$is positive and $A_{ex}^{pin-rot}$ is negative, $(A_{ex}^{pin-FM}-A_{ex}^{pin-rot})$ is always positive. Therefore, the interface energy density difference $\Delta\varepsilon$ is proportional to the quantity difference of $-\boldsymbol{z}$ and $+\boldsymbol{z}$ pinned spins, namely $\Delta n=(n_{+}-n_{-})$, as illustrated in Fig. 4(d) in the main text.

**Fig. S1. The X-ray diffraction results for the sample Ta (2 nm)/Ir_20_Mn_80_ (5 nm)/Co_40_Fe_40_B_20_ (1 nm)/MgO (2.5 nm)/Al_2_O_3_ (5 nm).** The black dotted lines indicate (111) orientation of IrMn.

**Fig. S2.** **The XAS and XMCD spectra of Mn and Fe at temperatures of (a) 2 K, (b) 100 K, (c) 200 K and (d) 300 K.** The total XAS is obtained by the sum of two spectra as $(I^{-}+I^{+})$, where $I^{-}$ and $I^{+}$ refer to the XAS spectra using left and right circularly polarized helical incident X-rays, respectively.

**Fig. S3. Element-specific Co XMCD hysteresis loops under varying field-annealing conditions.** (a-c) Hysteresis loops measured at 300 K for samples annealed under +1 T, -1 T, and no magnetic fields. (d-f) Corresponding loops measured at 10 K for the same annealing conditions.

Fig. S4. Mn XMCD hysteresis loops and corresponding Mn XMCD spectra. The XMCD loops are measured at 10 K and 300 K from samples annealed under (a)-(e) positive and (f)-(j) negative fields. $\boldsymbol{H}\mathbf{+}$ and $\boldsymbol{H}\mathbf{-}$ refer to the Mn XMCD spectra measured when applying positive and negative saturation magnetic fields, respectively. The XMCD intensity is normalized by the peak intensity of X-ray absorption spectra.

Fig. S5. Snapshots of the interfacial AFM in micromagnetic simulations of exchange bias. Spin configurations of AFM interfacial spins after annealing under (a) $\mathbf{-}\boldsymbol{z}$ fields and (b) $\mathbf{+}\boldsymbol{z}$ fields. The pinned spins are circled by white dashes. Red pinned spins align in $\mathbf{+}\boldsymbol{z}$ while blue pinned spins align in $\mathbf{-}\boldsymbol{z}$, with the other green area representing AFM rotatable spins.

## References

[1] J. De Clercq, A. Vansteenkiste, M. Abes, et al., Modelling exchange bias with MuMax3, J. Phys. D. Appl. Phys. 49 (2016) 435001.
[2] S. Peng, D. Zhu, W. Li, et al., Exchange bias switching in an antiferromagnet/ferromagnet bilayer driven by spin–orbit torque, Nat. Electron. 3 (2020) 757–764.
[3] J. De Clercq, A. Vansteenkiste, M. Abes, et al., Modelling exchange bias with MuMax3, J. Phys. D. Appl. Phys. 49 (2016) 435001.
